# Predictive value of carotid artery contrast-enhanced ultrasound combined with clinical indicators in ischemic stroke patients

**DOI:** 10.3389/fneur.2025.1660031

**Published:** 2025-10-30

**Authors:** Xinrong Song, Min Chen, Xiaona Wang, Huimin Niu

**Affiliations:** 1 Graduate School, Hebei Medical University, Shijiazhuang, Hebei, China; 2 Department of Ultrasound, Hebei General Hospital, Shijiazhuang, Hebei, China

**Keywords:** carotid artery, contrast-enhanced ultrasound, triglyceride-glucose index, LDL/HDL, ischemic stroke

## Abstract

**Objective:**

To assess the predictive significance of carotid artery contrast-enhanced ultrasound (CEUS) combined with the triglyceride glucose index (TyG index) and low-density lipoprotein (LDL) to high-density Lipoprotein (HDL) ratio in patients with ischemic stroke (IS).

**Methods:**

This study included 130 patients admitted to the hospital from June 2021 to June 2023. All patients underwent carotid artery CEUS and were divided into two groups: the ischemic stroke group (IS, *n* = 73) and the non-ischemic stroke group (NIS, *n* = 57). Baseline characteristics of both groups were analyzed and compared to identify differences. Logistic regression analysis was performed to determine independent risk factors for IS. Receiver operating characteristic (ROC) curves were constructed to assess the predictive performance of CEUS classification, TyG index, LDL/HDL ratio, and their combination.

**Results:**

Among the 130 patients, the IS group consisted of 73 individuals (53 males, 20 females), with an average age of 64.87 ± 10.55 years, while the NIS group included 57 patients (20 males, 37 females) with an average age of 63.12 ± 10.26 years. The IS group exhibited significantly higher CEUS classification, TyG index, and LDL/HDL ratio compared to the NIS group. Logistic regression analysis identified CEUS classification (*OR* 3.521, *95% CI* 1.731–7.161, *p* = 0.001), TyG index (*OR* 3.851, *95% CI* 1.129–13.142, *p* = 0.031), and LDL/HDL ratio (*OR* 2.957, *95% CI* 1.321–6.622, *p* = 0.008) as independent risk factors for IS. The areas under the ROC Curve (AUC) for predicting IS were 0.871 for CEUS classification, 0.766 for TyG index, and 0.735 for LDL/HDL ratio. The combined application of these indicators yielded an AUC of 0.910, which was significantly higher than that of any single indicator.

**Conclusion:**

CEUS classification, TyG index, and LDL/HDL ratio are useful predictors of IS. Their combined use significantly enhances predictive accuracy.

## Introduction

Stroke is a global health burden that jeopardizes human wellbeing and stands as the leading cause of mortality and disability worldwide (1). It is typically characterized by acute focal damage to the central nervous system caused by vascular causes, followed by neurological deficits. Stroke is broadly categorized into ischemic stroke (IS) and hemorrhagic stroke, with IS being the most prevalent form (2). IS is defined as an episode of neurological dysfunction resulting from focal cerebral, spinal cord, or retinal infarction (3). The early identification of risk factors that is therefore crucial for stroke prevention and management (4).

Atherosclerosis is a major cause of IS. The risk of IS lies not only in the narrowing of the carotid lumen caused by atherosclerotic plaques (5), but also in the stability of these plaques (6–8). Pelisek et al. (9) pointed out that intra-plaque neovascularization and inflammation are the two main features of vulnerable plaques, and plaque vulnerability increases the risk of stroke. Contrast-enhanced ultrasound (CEUS), a noninvasive imaging modality characterized by simplicity and high temporal and spatial resolution, can dynamically visualize the distribution and density of intraplaque neovascularization in real time and clearly delineate surface irregularities and ulcers on the plaque. This renders it valuable for assessing the stability of carotid artery plaques (10–12). However, the relationship between the degree of CEUS enhancement and IS requires further study.

Atherosclerotic diseases are also closely related to the levels of many biomarkers. The TyG index is calculated based on fasting triglyceride and fasting glucose levels. Studies have shown that an increase in the TyG index is associated with an increased risk of subclinical atherosclerosis, including coronary artery calcification and carotid plaque formation (13–15). Therefore, we speculate that the TyG index is also one of the risk factors for IS. Elevated low-density lipoprotein (LDL) levels and decreased high-density lipoprotein (HDL) levels are both considered risk factors for atherosclerosis and can be used to predict the occurrence and development of atherosclerosis (16–19). Enomoto et al. (20) conducted an 8-year epidemiological follow-up study and found that the LDL/HDL ratio is more valuable in predicting atherosclerosis than LDL or HDL alone. Therefore, the LDL/HDL ratio was selected to investigate its predictive utility for stroke.

In summary, both the TyG index and LDL/HDL ratio are intimately associated with the progression of carotid atherosclerosis, yet their correlation with IS remains inadequately explored. In this study, CEUS combined with the TyG index and LDL/HDL ratio is proposed to create a non-invasive prediction model. This model will integrate multimodal fusion of blood biomarkers (reflecting macroscopic metabolic derangements) and imaging parameters (reflecting microvascular function), synthesizing information across multiple pathophysiological (i.e., metabolism, lipid profile, vascular function), to enable a more comprehensive and precise stratification of IS risk. By analyzing the predictive value of CEUS combined with the TyG index and LDL/HDL for IS, new ideas and bases for the clinical prevention and treatment of IS are provided.

## Methods

### Patients

This retrospective study was approved by the Medical Ethics Committee of Hebei General Hospital, and the Institutional Review Board of this institution waived the requirement for informed consent. A retrospective analysis was conducted on 130 inpatients who underwent carotid artery Contrast-Enhanced Ultrasound (CEUS) in the Department of Ultrasound of Hebei General Hospital between June 2021 and June 2023. The inclusion criteria were: (1) age>40 years, (2) hospitalized patients with carotid plaque, (3) conscious patients capable of cooperating with carotid plaque ultrasonography, (4) completion of brain CT or MRI examinations. The exclusion criteria were: (1) extensive carotid plaque calcification, (2) hypersensitivity to ultrasond contrast agent, (3) diagnosis of malignant tumors, (4) severe malnutrition; and (5) comorbidity with other cerebrovascular disorders. All patients were stratified into the ischemic stroke group (IS group, *n* = 73) and the non-ischemic stroke group (NIS group, *n* = 57) based on their clinical manifestations and brain CT or MRI findings within 6 months.

### Carotid artery CEUS

**Figure 1:**
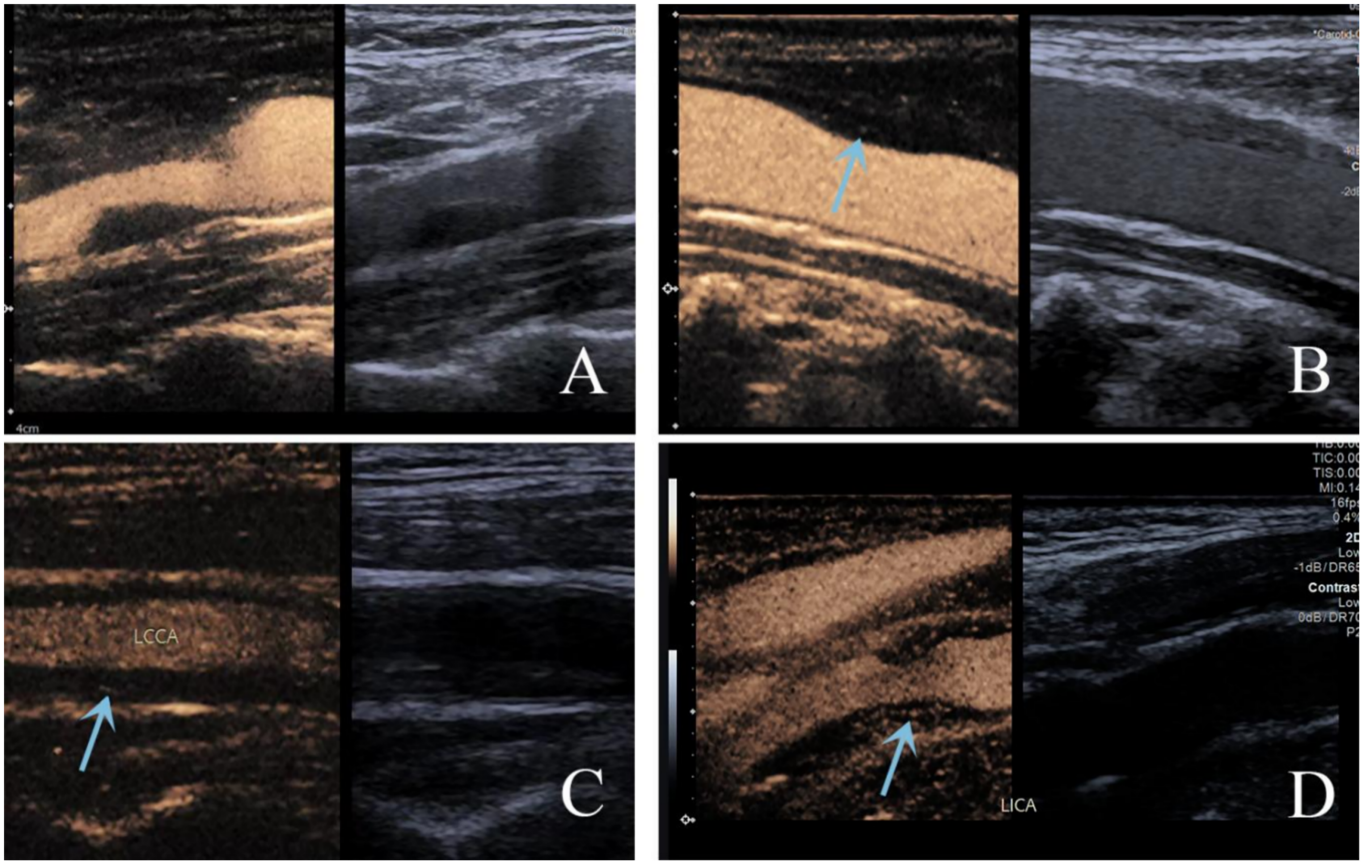
Standardized grading criteria for the degree of contrast enhancement within plaques on ultrasound: A Score 0: no intraplaque enhancement; B score 1: punctate intraplaque enhancement; C score 2: intermediate between scores 1–3, characterized punctate and 1–2 short linear of enhancement within the plaque; D score 3: linear intraplaque enhancement, penetrating or involving the majority of the plaque, orvisible signs of blood flow.

Ultrasound examinations were performed using a Siemens Acuson Sequoia color Doppler unit with an L9-3 probe. The contrast agent SonoVue (Bracco, Milan, Italy) was administered via an antecubital vein. Dynamic contrast-enhanced images were acquired to evaluate the distribution of the contrast agent within the plaque for CEUS classification. Plaques were classified as follows: Score 0: no intraplaque enhancement; score 1: punctate intraplaque enhancement; score 2: intermediate between scores 1–3, characterized punctate and 1–2 short linear of enhancement within the plaque; score 3: linear intraplaque enhancement penetrating or involving the majority of the plaque, or visible signs of blood flow (21) (Figure 1). Standardization and Blinding Procedures: Plaque grading was independently conducted by two senior ultrasound physicians with substantial CEUS expertise. Both examiners were blinded to the clinical data and group assignment. In the event of discrepancies, consensus was achieved via discussion. This ensured standardized plaque evaluation and minimized assessment bias. The inter-rater agreement for plaque grading between the two independent raters was assessed using the Kappa statistic, which yielded a value of 0.76 (95% CI 0.70–0.86), indicating good agreement between the raters.

### Collection of clinical indicators

Baseline characteristics and risk factors, including age, sex, hypertension, diabetes mellitus, smoking status, fasting glucose, fasting triglyceride, low-density lipoprotein (LDL), and high-density lipoprotein (HDL) levels, were collected. The TyG index was calculated using the formula: ln [fasting triglycerides (mg/dL) × fasting glucose (mg/dl)/2] (22).

### Statistical analysis

All statistical analyses were performed using IBM SPSS Statistics (version 27.0). Continuous data are presented as mean ± standard deviation or median (interquartile range, P25 to P75) and were analyzed using the independent t-test or the Mann–Whitney U test. Categorical data are summarized as frequency (n) or percentage (%) and were compared using the chi-square test. Multicollinearity among predictors was assessed by calculating the variance inflation factor (VIF). Independent risk factors for IS were identified using multivariable Logistic regression The predictive performance of the CEUS classification combined with the TyG index and the LDL/HDL ratio for IS was evaluated using a receiver operating characteristic curve (ROC). The Delong test was utilized to compare the areas under the ROC curves (AUCs) of the combined model and each single index model pairwise. A two-sided *p*-value of less than 0.05 was considered statistically significant.

## Results

### Univariate analysis

A total of 130 patients are enrolled, with 73 in the IS group and 57 in the NIS group. The results of the univariate analysis comparing the two groups are presented in Table 1. Statistically significant differences (*p* < 0.05) were observed in sex (*p* < 0.01) and hypertension (*p* = 0.01), suggesting a higher prevalence of IS among male patients with hypertension. Furthermore, the IS group demonstrated significantly higher values for CEUS classification, the TyG index, and the LDL/HDL ratio compared to the NIS group (all *p* < 0.05). In contrast, no statistically significant differences were noted between the two groups regarding age (*p* = 0.343), diabetes (*p* = 0.184), or smoking history (*p* = 0.219).

**Table 1:** Comparison of characteristics between the IS group and the NIS group.

Lems	IS (*n* = 73)	NIS (*n* = 57)	*t/X^2^/z*	*p*
Age (year, mean ± SD)	64.87 ± 10.55	63.12 ± 10.26	0.95	0.343
Male (*n*, %)	53(72.6%)	20(35.0%)	18.30	<0.01
Hypertension (*n*, %)	50(68.4%)	22(38.5%)	11.58	0.001
Diabetes (*n*, %)	28(38.3%)	16(28.0%)	1.76	0.184
Smoking (*n*, %)	30(41%)	17(29%)	1.12	0.219
TyG, Median (IQR)	8.75(8.32,9.00)	8.08(7.92,8.55)	−5.41	<0.01
LDL/HDL, Median (IQR)	3.31(2.52,3.50)	2.29(1.91,2.68)	−4.79	<0.01
CEUS (*n*, %)			59.34	<0.001
Grade 0	4(5.5%)	25(43.9%)		
Grade 1	7(9.6%)	21(36.8%)		
Grade 2	28(38.4%)	9(15.8%)		
Grade 3	34(46.6%)	2(3.5%)		

### Multivariate analysis

Multicollinearity analysis revealed that the VIF for all variables was belpw 2, indicating no significant collinearity and thereby confirming the statistical robustness and independence of the predictors. Sex, hypertension, CEUS classification, TyG index, and LDL/HDL ratio were included in the multifactorial logistic regression analysis, with the occurrence of IS as the dependent variable. The results demonstrated that CEUS classification (OR 3.521, 95%CI 1.731–7.161, *p* = 0.001), TyG index (OR 3.851, 95%CI 1.129–13.142, *p* = 0.031), and LDL/HDL ratio (OR 2.957, 95%CI 1.321–6.622, *p* = 0.008) were the independent risk factors for IS (Table 2).

**Table 2:** Multivariate logistic regression analysis of influencing factors for IS.

Independent variable	*β*	*SE*	*Wald*	*p*	*OR*	*95%CI*	
CEUS	1.259	0.362	12.074	0.001	3.521	1.731	7.161
TyG	1.348	0.626	4.636	0.031	3.851	1.129	13.142
LDL/HDL	1.084	0.411	6.951	0.008	2.957	1.321	6.622
Sex	−0.936	0.577	2.632	0.105	0.392	0.127	1.215
Hypertension	0.712	0.636	1.251	0.263	2.038	0.585	7.091

### Predictive efficacy

**Figure 2:**
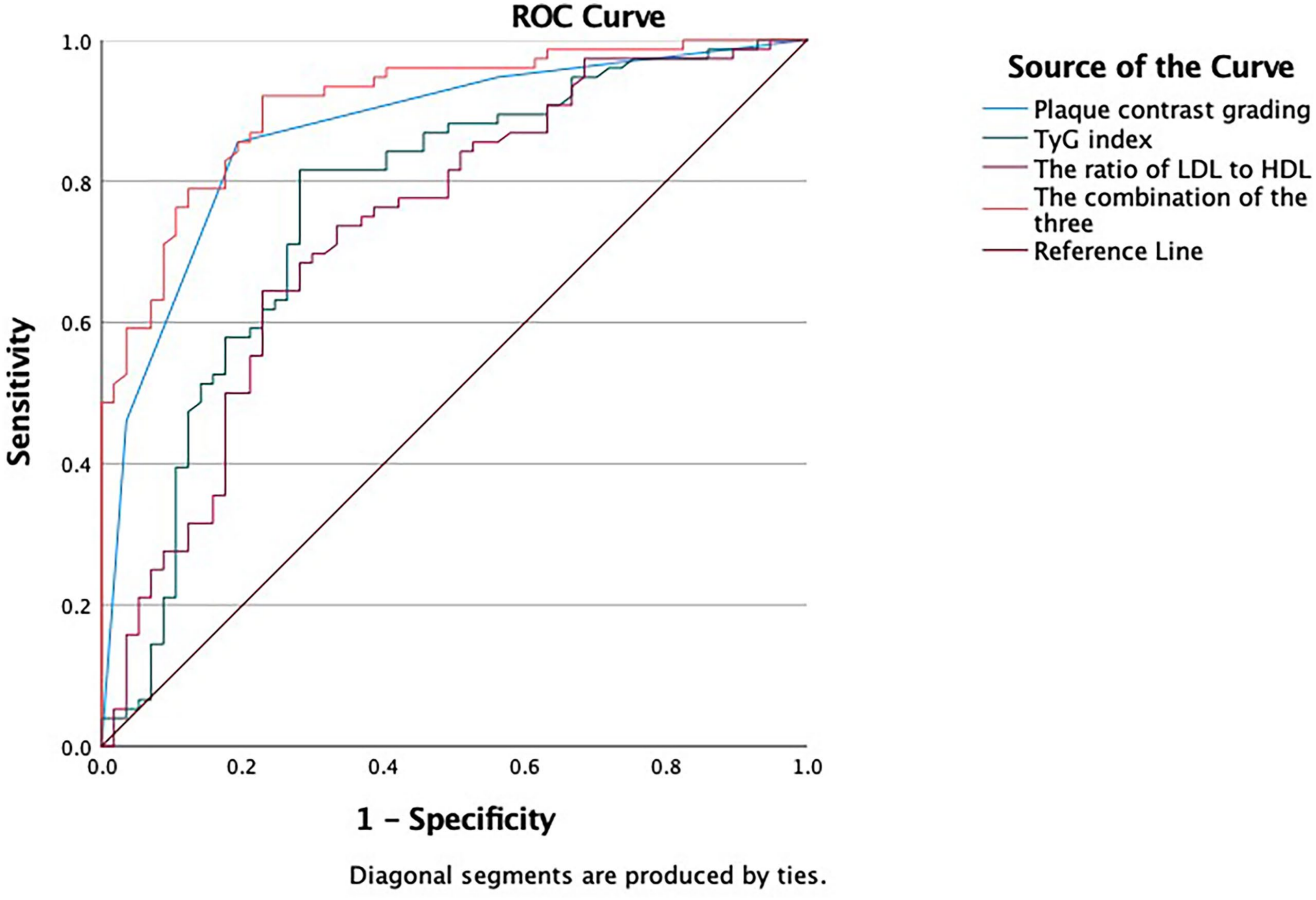
ROC curve depicting the predictive efficacy of CEUS classification, TyG index, LDL/HDL ratio, CEUS classification+TyG index+ LDL/HDL ratio for IS.

The areas under the ROC curve (AUC) for CEUS classification, the TyG index, the LDL/HDL ratio, and the combined model (CEUS classification+TyG index+LDL/HDL ratio) were 0.871 (95% CI 0.809–0.933), 0.766 (95% CI 0.680–0.851), 0.735 (95% CI 0.647–0.822), 0.910 (95% CI 0.862–0.958), respectively (Figure 2 and Table 3). The Delong test indicated statistically significant differences in the AUC values among these four models, suggesting that the combined application of these predictors yields superior performance compared to any single indicator alone (Table 4). Notably, the combined model exhibited higher sensitivity and specificity (0.867, 0.879) than CEUS classification alone (0.823, 0.846), the TyG index alone (0.741, 0.728),or the LDL/HDL ratio alone (0.705, 0.712).

**Table 3:** The predictive efficacy of each indicator for IS.

Targets	AUC	*95CI*%	Sensitivity	Specificity
CEUS	0.871	0.809, 0.933	0.823	0.846
TyG	0.766	0.680, 0.851	0.741	0.728
LDL/HDL	0.735	0.647, 0.822	0.705	0.712
Combination	0.910	0.862, 0.958	0.867	0,879

**Table 4:** The result of delong test.

Comparison combination	CEUS	TyG	LDL/HDL
P(Delong test)	0.034	<0.001	<0.001

## Discussion

Ischemic stroke (IS) is a prevalent stroke subtype with acute onset, rapid progression, and poor prognosis, highlighting the critical importance of early identification of high-risk patients (23). A growing body of evidence has confirmed that the rupture of vulnerable carotid plaques is a pivotal trigger for ischemic cerebrovascular events (6, 8). Extensive research has demonstrated that neovascularization within plaques is a robust predictor of IS in patients with carotid artery plaques (24–27). Currently, CEUS is the most widely used clinical method for assessing atherosclerosis and offers rapid imaging capabilities. It enables real-time dynamic observation of intra-plaque neovascularization via intravenous injection of microbubble-containing contrast agents. With technological advancement, CEUS has emerged as an increasingly favored ultrasound modality owing to its lack of radiation, renal safety (superior to CT/MRI contrast agents), and cost-effectiveness. Numerous studies have validated that CEUS classification correlates well with plaque microvessel density (MVD) and exhibits high specificity (28). Our findings show that CEUS classification is an independent risk factor for IS, with a significantly higher prevalence in the IS group than in the NIS group. Specifically, in the IS group, approximately 85.0% of patients had a CEUS classification of 2–3, a proportion notably higher than that in the NIS group. These results align with other studies (29, 30), collectively confirming the predictive value of CEUS classification for IS occurrence.

The TyG index, calculated from fasting glucose and fasting triglyceride levels, is recognized as a reliable indicator of insulin resistance (31, 32). Insulin resistance induces chronic inflammation and endothelial dysfunction, promoting foam cell formation, atherosclerosis, and the development of vulnerable plaques (33, 34). It has been established that a higher TyG index correlates with an increased incidence of cardiovascular disease and stroke (35, 36), and patients with IS exhibiting a higher TyG index face elevated risks of stroke recurrence and mortality (22, 37). The present study demonstrates that the TyG index in the IS group is significantly higher than that in the NIS group, which aligns with previous research findings (26, 38). Collectively, these results indicate that the TyG index is associated with an augmented risk of IS and serves as an independent risk factors for predicting IS.

The findings of this study suggest that the LDL/HDL ratio is a highly valuable indicator for predicting IS and constitutes an independent risk factors for IS occurrence, consistent with the research by Ciplak et al. (39). A number of existing studies have revealed that elevated cholesterol levels are closely linked to an increased risk of IS, and hypercholesterolemia is recognized as a modifiable vascular risk factors (40–42). LDL and HDL are the main lipoproteins responsible for cholesterol transport; elevated LDL and decreased HDL are crucial risk factors for IS (43). Another study has shown that the formation of carotid artery plaques increases with a rising LDL/HDL ratio (20) and that the LDL/ HDL ratio provides more information for predicting atherosclerosis than LDL or HDL alone, particularly in individuals with diabetes and dyslipidemia (44). Mechanistically, an increased LDL/HDL ratio may be associated with the formation of a necrotic core due to sustained lipid accumulation within plaques.

This study also identifies statistically significant differences between the two groups in terms of sex and hypertension, consistent with well-established studies. However, no statistically significant differences were observed in age, history of diabetes mellitus, or smoking history, which deviates from currently recognized clinical perspectives. This discrepancy is hypothesized to be related to the relatively small sample size of this study. Logistic regression analysis indicates that sex and hypertension were not independent risk factors for IS in our cohort, which is inconsistent with the results of some other studies (45, 46). Potential reasons may include that (1) the effect of sex may beconfounded by other risk factors, and (2) blood pressure instability might have influenced the assessment of hypertension. Nevertheless, these well-recognized risk factors still hold fundamental significance for the clinical identification of individuals at risk of IS.

Our study has several limitations: (1) It was conducted at a single center with a limited sample size, which may constrain the generalizability of the results. External validation via multi-center studies with larger cohorts is required. (2) The study population primarily consisted of Chinese participants. Given that multiple studies have documented differences in metabolic disease profiles, stroke subtypes, and vascular biology among different races (47, 48). Therefore, caution is required when extending the results to other racial groups. (3) Our dataset lacked information on potential confounding factors, such as statin use and antiplatelet drug administration. Additionally, fasting blood glucose and triglyceride levels could have been influenced by various interventions prior to hospital admission, a factor that could not be fully controlled in this study. These limitations may have introduced selection bias and affected the precision of the risk factor analysis. Future research should involvet larger-scale, multicenter studies to validate and refine the model, thereby enhancing its accuracy and general applicability.

## Conclusion

CEUS classification, the TyG index, and the LDL/HDL ratio are independent risk factors for IS and demonstrate good predictive value. Their combined application can improve the sensitivity and specificity of IS prediction. Compared with serum indicators such as the TyG index and LDL/HDL ratio—which only reflect metabolic and lipid levels—CEUS offers irreplaceable advantages by providing real-time dynamic microcirculation information. Therefore, we propose that a combined model incorporating CEUS classification, the TyG index, and the LDL/HDL ratio holds potential as an effective tool for risk prediction in IS patients. This model offers a reliable basis for early identification of high-risk individuals, risk stratification, and personalized diagnosis and treatment strategies.

## Data Availability

The original contributions presented in the study are included in the article/supplementary material, further inquiries can be directed to the corresponding author.
