# Correction: Predictive value of carotid artery contrast-enhanced ultrasound combined with clinical indicators in ischemic stroke patients

**DOI:** 10.3389/fneur.2025.1755097

**Published:** 2025-12-08

**Authors:** Xinrong Song, Min Chen, Xiaona Wang, Huimin Niu

**Affiliations:** 1 Graduate School, Hebei Medical University, Shijiazhuang, Hebei, China; 2 Department of Ultrasound, Hebei General Hospital, Shijiazhuang, Hebei, China

**Keywords:** carotid artery, contrast-enhanced ultrasound, triglyceride-glucose index, LDL/HDL, ischemic stroke

In the published article, there was an error the funder “[Hebei Province Health Commission Medical Research Project Plan], [Grant Number: 20220881] to [Huimin Niu]” was erroneously omitted. The corrected statements appear below.

The original version of this article has been updated.

